# Reflections on global policy documents and the WHO's infant feeding guidelines: lessons learnt

**DOI:** 10.1186/1746-4358-5-18

**Published:** 2010-10-26

**Authors:** Astrid Blystad, Penny van Esterik, Marina M de Paoli, Daniel W Sellen, Sebalda C Leshabari, Karen Marie I Moland

**Affiliations:** 1 Department of Public Health and Primary Health Care, University of Bergen, Norway; 2 Centre for International Health, University of Bergen, Norway; 3 Department of Anthropology, York University, Canada; 4 Fafo Institute for Applied International Studies, Oslo, Norway; 5 Department of Anthropology, University of Toronto, Canada; 6 Muhimbili University of Health and Allied Sciences (MUHAS), Tanzania; 7 Faculty of Health and Social Sciences, Bergen University College, Norway

## Abstract

As the papers in this thematic series have illustrated, the postnatal prevention of mother to child transmission of HIV (PMTCT) strategy has struggled with lack of local relevance. In an attempt to increase our understanding of the great dissonance between the policy intention and the experiences of the participants in concrete PMTCT programmes, we will in these concluding remarks draw upon writings in institutional ethnography. Through the concept of 'global texts' we reflect upon the scientific and ideological underpinnings of the WHO policy guidelines on HIV and infant feeding, and the influence that this policy has had across multiple local settings. The particular impact of the global postnatal PMTCT policy guidelines on the position of breastfeeding lies at the core of the discussion.

## Introduction

We close this thematic series titled 'HIV and infant feeding: lessons learnt and ways ahead' by reflecting first, in the present paper, on the lessons learnt during the past decade of PMTCT programme implementation and then, in the subsequent paper, by briefly discussing ways ahead. With an attempt to grasp the meaning and significance of the unexpected and disturbing outcomes of previous PMTCT infant feeding guidelines documented in the papers of this issue we will reflect on them as part of a growing body of policy and implementation documents operating on the global arena. A brief discussion of the production, ideological underpinnings and effects of such documents can, we believe, enhance our understanding of some of the central dynamics at work in efforts to prevent MTCT. The grounded qualitative studies of PMTCT clients and providers presented in this issue reveal the complex and often troubled relationship between such policy guidelines and the 'local', and their particular cultural and political underpinnings and historical provenance.

### The PMTCT guidelines as global texts

Dorothy Smith has written extensively about the production of what she calls 'global texts' and documents in organisations such as the UN and the World Bank. Her writings reveal how such texts make the co-ordination of thought and action across multiple local settings and times possible [1]. The policy documents are produced by people in one setting while read and implemented by people in multiple other local settings [1]. From a particular text or document produced globally it is possible to trace sequences of action locally, and one can identify where and how the text attempts to 'standardize action' and coordinate people's activities across multiple local sites.

#### Global texts - local lives

Smith argues that 'global documents', in the sense we are talking about here, are constructed through processes of consensus building and produce texts that appear as shared and that create a common ground [1]. The processes that produce such global documents that are supposed to work across multiple settings will necessarily have to suppress divergent perspectives and create discursive entities and a conceptual space in which texts can be related to one another without any reference to people [1]. Drawing upon Smith's insights, we will argue that it is the capacity of such texts or documents to exist beyond particular times, places and peoples that underlies the many and complex challenges that the PMTCT programming has encountered.

Smith holds that it is not enough to use global texts as 'sources of information' [1]. Rather they should be seen as they enter into people's local practices and by how they mediate, regulate and authorize people's activities. In this issue we have tried to do this; we have tried to trace how the WHO infant feeding guidelines enter into people's local lives and practices and how they manifest themselves across different settings. Through diverse studies it has become clear that the guidelines lack reference to people's lives, and to breastfeeding as embodied knowledge and as a culturally embedded practice. In order to facilitate coordination and standardization, the ontological ground of breastfeeding is left vague and undefined.

This disconnect is not a trivial matter as it obscures the contextual factors that limit the capacity for uptake and adherence by women as the intended beneficiaries of the programme.

#### Cultural/ideological underpinnings of global texts

To facilitate the ambition of global relevance, international policy documents or global texts thus appear as 'existing beyond culture and time'. Such framing of international policy documents has been increasingly criticized. Abu-Lughod [2] for instance writes that the discourse surrounding international documents needs to be fundamentally confronted with the implicit construction of such documents as 'a-cultural' and 'universal', and for the reification and freezing of what is deemed to be 'the cultural'. She argues that the 'cultural' in such texts is commonly represented as the 'non-Western'. Engle Merry [3] in the same way demonstrates how the international language of human rights - which commonly makes up an ideological basis of global documents or texts - is not neutral. It is fundamentally cultural and reflects the particular values found in secular global modernity characterized by concepts of autonomy, individuality and equality.

The global PMTCT guidelines of the WHO have a very clear basis in human rights and human development thinking as well as in modern scientific thinking, more concretely in biomedical thinking. Biomedical discourse related to the 2001 WHO infant feeding guidelines [4] was based on scientific evidence that HIV can pass from mother to child during pregnancy, birth and breastfeeding, and that there are dangers related to mixed feeding in an HIV context. The human rights discourse has called for the rights to know (e.g. women's rights to receive information about the dangers of transmission of HIV through breast milk), and the right to choose (e.g. between different infant feeding options). The papers in this issue provide disturbing examples of how application of both sets of ideas/knowledge have failed to provide effective guidance for many HIV-infected women.

The implications of a biomedical focus on HIV infection transmitted from mother to child, rather than on infant survival more broadly has been pointed out as a key challenge. This has been powerfully demonstrated in the study from Kwa Zulu Natal published in 2007 by Coovadia and colleagues [5]. The narrow focus on HIV prevention seems to have overshadowed the long standing local knowledge and the scientific evidence of the superior role of breastfeeding in securing infant survival. It has also overshadowed the cultural and social importance of breastfeeding [6], and the intricate web of economic, practical, social, and cultural restrictions that are placed on women in an infant feeding context.

Being intrinsically individualistic, rights-based approaches tend to underestimate and obscure the relational bases of women's decision making [2]. As we have seen in the papers in this issue, the mother who enjoys the autonomy and the capability to weigh the risks and benefits of the various infant feeding options against each other, and carefully makes her decision is rare. Rather, women commonly live their lives as parts of larger networks of people who participate in each other's decisions on child health in general and on infant feeding in particular.

Another problematic feature of the biomedical and rights-based discourses informing the guidelines is that they do not directly address the immense constraints imposed by poverty. The PMTCT guidelines are deployed within - but do not acknowledge explicitly - a global landscape characterized by gross inequity. Although the limitations on choice of infant feeding method implied by resource constraints were recognized by the invention and adoption of the concept of 'AFASS criteria', the concept remains vague and has proven to be very difficult to operationalize and measure. The 2001 infant feeding guidelines [4] were read and employed as if the stated infant feeding options implied actual or true options of choice irrespective of available resources. A 'choice in non choice situations'-like scenario emerged; a scenario with dubious ethical underpinnings. In actual life poverty clearly leaves the vast majority of HIV-infected women with little or no option regarding replacement feeding with commercial infant formula.

In line with Abu Lughod [2] we ask how reliance on particular knowledge and on a particular vernacular - in this case grounded in biomedical and human rights thinking - may colour the representation of the challenge at hand, and simultaneously foreclose certain solutions to the challenge. By drawing on a biomedical and rights based understanding without a link to locally grounded experience, both the understandings of the challenge and the proposed solutions or interventions to counter the challenge may turn out to be highly problematic. Powerful evidence of the unintended and undesirable outcomes of such mismatches has been presented in this thematic issue.

## Concluding remarks

The papers in this issue have focussed on the challenges and failures of previous efforts to manage infant feeding practices by HIV positive mothers and postnatal PMTCT counsellors. Taken together, the results of the work have demonstrated how 'global texts' - such as the growing family of PMTCT guidelines - articulate with local exigencies, and are acted upon by individuals and communities at concrete moments in time. Critics of global policy texts argue that their impact or their regulatory strength ultimately depends on their cultural legitimacy and on their embodiment in local culture [1,3]. The major theme that runs through the preceding papers is of the confusion the previous guidelines have instigated for both mothers and frontline health care workers across diverse settings because of their lack of local relevance and applicability.

At the simplest level the poor applicability of the earlier guidelines to local needs and realities has meant that they have not been able to facilitate the changes in infant feeding practices that they aimed for. In addition, the social and cultural distance between the producers of the guidelines and its many recipients has generated a sense of helplessness, confusion, guilt and fear among the ones involved in the intervention. Women have been unable to adhere to the infant feeding recommendations presented in the guidelines with subsequent emotional stress and fear of harming their infants. PMTCT counsellors have reported uncertainty and loss of faith in their work in infant feeding after experiences with the large numbers of non-adherent mothers. Many are indeed worried that the confusion about infant feeding has reduced the public trust in nursing as a profession [6].

The articles in this issue have revealed the adverse effects produced during attempts at implementing the guidelines, and add important evidence to the growing documentation of a disturbing impact of global policy texts that are out of touch with local lives. Hope lies in the recent shifts in the PMTCT guidelines, shifts that imply a retreat to the promotion of infant feeding practices that are located far closer to customary feeding patterns.

## Competing interests

The authors declare that they have no competing interests.

## Authors' contributions

AB and KMIM wrote the first draft of the paper. PVE was a key person during the workshop and wrote a summing up paper that was drawn upon in these concluding remarks. MMDP and DWS contributed to subsequent drafts. SCL contributed in the writing process. All authors approved the final paper.
